# Modeling and Analysis of Anomalies in the Network Infrastructure Based on the Potts Model

**DOI:** 10.3390/e23080949

**Published:** 2021-07-25

**Authors:** Andrzej Paszkiewicz

**Affiliations:** Department of Complex Systems, The Faculty of Electrical and Computer Engineering, Rzeszow University of Technology, al. Powstańców Warszawy 12, 35-959 Rzeszów, Poland; andrzejp@prz.edu.pl

**Keywords:** Potts model, Ising model, phase transitions, anomalies, complex systems, IT networks

## Abstract

The paper discusses issues concerning the occurrence of anomalies affecting the process of phase transitions. The considered issue was examined from the perspective of phase transitions in network structures, particularly in IT networks, Internet of Things and Internet of Everything. The basis for the research was the Potts model in the context of IT networks. The author proposed the classification of anomalies in relation to the states of particular nodes in the network structure. Considered anomalies included homogeneous, heterogeneous, individual and cyclic disorders. The results of tests and simulations clearly showed the impact of anomalies on the phase transitions in the network structures. The obtained results can be applied in modelling the processes occurring in network structures, particularly in IT networks.

## 1. Introduction

Network structures are common in natural and artificial environments. Both natural and artificial ones belong to the group of complex systems [1]. These systems are characterised by such properties as: self-similarity, power law, self-adaptation, etc. [2]. Most of them were examined and analysed in the context of natural, economic, social, political, and technical sciences [3,4]. Thus far, due to the complexity of the problem of complex system analysis, it has not been possible to develop a single model that takes into account all the features of these systems. Therefore, at this stage of the research, complex systems are analysed in the context of an individual or a limited number of features. The occurrence of the phase transition phenomenon in complex systems is one of their properties. This phenomenon is related to a rapid change in the value of significant parameters of a given object/system/structure, which usually leads to a change in its properties. Thus, this phenomenon is critical. One of the models allowing the analysis of phase transitions is the Ising magnetism model [5,6,7,8,9,10,11] and its generalisation in the form of the Potts model [12,13,14]. The potential of this model in studying the behaviour and properties of IT structures was presented in the author’s earlier study [15]. The properties and implementation of these models confirm their wide area of application. They are ideally suited to the study of phenomena of change in the properties of a given system in the fields of physics and electronics, but also to the analysis of economic and social relations [16,17,18]. Of course, they are also used in widely understood IT systems. However, typically, these models are used in these cases to model the interaction between nodes in wired, wireless or social networks [19,20,21,22]. It is important to note that in [23], the authors also analysed the spread of malware based on the Ising model. This approach confirms the potential of the Potts and Ising model to analyse IT systems in terms of the properties characteristic of complex systems. Nevertheless, so far, these models have not been used to model the phenomena of anomalies and disturbances in the structures of IT networks more generally.

Anomalies or disorders occurring in the studied environment have attracted the interest of scientists from various fields [24]. The aim of the aforementioned paper was to determine the causes of the formation of such phenomena, as well as to determine the possible effects and consequences of their presence. In addition, processes related to the occurrence of this type of phenomena have also been subject to research. An important aspect of the research is to determine the nature of these phenomena, i.e., whether they are random, regularly repeated, individual (relate to a single anomaly) or group anomalies, and whether they occur locally or in the whole environment. Conducted research covers mostly issues related to the security of IT systems [25]. In this context, systems are tested in terms of work under normal conditions and then under abnormal conditions. On this basis, the system reference states are defined. By analysing possible discrepancies, it is possible to detect attacks on IT but also on IoT and IoE networks. This applies to both computer systems as well as intermediate devices such as switches, routers and end-devices including sensors, and other active elements. In the scientific literature, a great deal of papers can be found on anomaly detection based on network traffic analysis using, among others, artificial intelligence, machine learning, statistical analysis or properties of complex systems [26,27,28,29,30]. However, there is an apparent lack of models to analyse the processes that take place when such events occur and the phenomena that accompany them. Therefore, the main objective of this article is to present the possibility of modelling the inter-node interaction in a network structure, taking into account different types of disturbances. It should be noted that this paper does not include analysis of the causes of anomalies in network structures, only their impact on the phase change process, as well as the course of changes taking place in the structure of IT networks. The paper will address issues related to the random occurrence of disturbances/anomalies in the network structure, as well as cyclic phenomena. The research was carried out on the basis of the Potts model.

The rest of the paper is organised as follows: Section 2 is a reference to the essence of phase transition and the assumptions of the Potts model and presents the order parameter as a tool to illustrate the phase transition process. Section 3 introduces the concept of anomalies/disturbances in the network structure and presents their classification, taking into account changes in the states of individual nodes. Section 4 covers the results of simulations performed and their analysis. Potential areas of application of the developed solution are presented in Section 5. The paper ends with a summary of the obtained research results and conclusions.

## 2. Phase Transition and the Potts Model

In order to refer to a phase transition, the concept of phase should be defined at the very beginning. Of course, numerous definitions exist depending on the field in which it is used [5,6]. However, for the purposes of this publication, the following concept was adopted.

Each node can be in one of the allowed states at any given time. Therefore, the values assigned to each node correspond to the individual states of the set $s=\left\{s_{i},i\in N\right\}$. The set *s* is a finite set. As a result of the interaction of neighbouring nodes, the state of a node may change. Frequent changes in the states of individual nodes may indicate instability in the entire network infrastructure. Therefore, the duration of continuous changes in node states across the network infrastructure will be referred to as the unstable phase. Such a phase is characterised by high dynamics, which is expressed by a change in the value of the order parameter. This parameter will be described in more detail later in this section. In contrast to the unstable phase, the network can be in a stable (ordered) phase. Then, the value of the mentioned order parameter reaches a constant value. Thus, the transition of a structure/system from one phase to another will be called a phase transition. It can therefore be said that the individual phases are separated from each other by interfacial surfaces, called phase boundaries, where a significant change in the properties of a given system/structure occurs. Such a boundary can be observed by analysing the variation in the value of the order parameter. Of course, in this case, we are talking about a boundary in parametric terms, not spatially. External factors can change the state of individual nodes as well as the entire infrastructure. This property has been used in this paper to model, as well as analyse, different types of network anomalies. 

In order to define the phase transition in network structures, let us assume that *G = {V, E}* is the network structure, where *V* is the set of vertices (nodes) and *E* the set of edges. However, the system remains in one of the global *S* states (stable or unstable) corresponding to a given phase. Then, the phase transition indicating the transition of the network structure *G* from the state *S* to the state *S’* is described by:

(1) $$G^{s}\overset{f}{\to }G^{s^{\prime }}$$

As a result of the transition of the network structure from the *S* state to the *S’* state, macroscopic changes occur, which are closely related to changes in the *G* structure properties. It should be noted that, very rarely, a change in a single node or edge condition directly contributes to the phase transition of the entire structure. However, it may be the initial trigger of the transformation process. A good example of a structural phase transition is the operation of the Girvan–Newman algorithm [31]. This algorithm is usually used to extract a community in graphs by successively removing edges from the original graph. In this case, we are talking about the phase of network cohesion and the phase of inconsistency. However, this algorithm does not refer to the states of individual nodes; therefore, the Potts model was used for the needs of the research [12,13,14]. This model is a generalisation of the Ising model of magnetism [7]. The classic Ising model is based on the assumption that each node (spin) can take one of two values, +1 or −1, i.e., $s_{i}\in \left\{-1,1\right\}$. The energy of any state is given by the Hamiltonian function [32]:

(2) $$H=-\sum_{\langle i,j\rangle }J_{ij}s_{i}s_{j}-B\sum_{i}s_{i},$$

where *J*_ij_ is the coupling constant between the *i*-th and *j*-th nodes, while *B* is the outer magnetic field. However, for the purposes of this paper, it was assumed that there could be any finite number of states in the network structure. These states may refer to various parameters, such as the opinion of a given node, the state of infection, the state of overload, etc. Therefore, it was assumed that the Potts model correlates better with these assumptions. It allows more states to be considered (two states are assumed in the Ising model). Furthermore, given the nature of IT networks, the part of the expression relating to the impact of an external field has been omitted. In addition, the coupling constant *J* for these considerations may take the value equal to one. Then, expression (2) takes the form:

(3) $$H=-\sum_{\langle i,j\rangle }s_{i}s_{j}.$$

However, to describe the relationship between the nodes *s*_i_ and *s*_j_, expression (3) is presented as:

(4) $$H=-\sum_{\langle i,j\rangle }\delta \left(s_{i}s_{j}\right),$$

where $\delta \left(s_{i}s_{j}\right)$ corresponds to the relationship between the states of two nodes and it is Kronecker delta, which equals one whenever *s*_i_ = *s*_j_ and zero otherwise. Each of the nodes can take one of the q values from the set of available states $s_{i}\in \left\{s_{i}^{1},s_{i}^{2},\ldots ,s_{i}^{q}\right\}$ at a given moment. 

When considering two separate states *s* and *s’*, as well as the probability of their occurrence as p(s) and $p\left(s^{\prime }\right)$, the relative probability that the system is in two states is:

(5) $$p=\frac{\;p\left(s^{\prime }\right)}{p\left(s\right)}=\frac{e^{\beta H\prime }}{e^{\beta H}}\;,$$

where β=1/kT parameter specified by the absolute temperature *T* and the Boltzmann constant *K*, *H* is the Hamiltonian of state *s*, and *H’* is the Hamiltonian of state *s’*. In ICT networks, the temperature parameter cannot be taken into account because such a value does not exist. Of course, this is a certain simplification, but a necessary one, and one that arises from the characteristics of IT systems. In this case, the change of states from *s* to *s’* occurs when $p\left(s^{\prime }\right)/p\left(s\right)\geq 1$ and p(s), $p\left(s^{\prime }\right)$ denote the relative probability that the system is in these particular states. Therefore, the probability of a node state change occurring can be briefly written as $p_{flip}=max\left(1,p\right)$.

**Figure 1:**
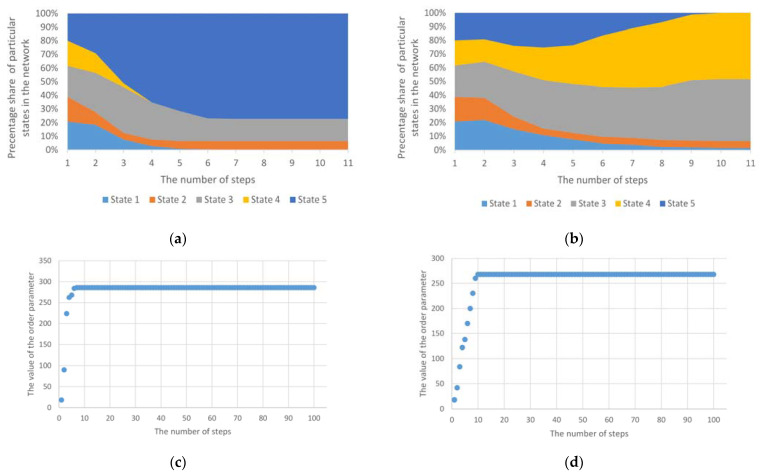
The course of changes of 5 states for the mechanism based on the Potts model: (**a**) standard (classic) approach; (**b**) approach with a threshold value; (**c**) values of the order parameter for the classic Potts model; (**d**) values of the order parameter for the Potts model, taking into account the threshold value.

The operation of two exemplary mechanisms based on the model described above for five different initial node states is presented in Figure 1. The first refers to the standard operation of the model, while the second presents the behaviour of the network structure in the case of introducing a threshold determining the change in the status of individual nodes. The results presented included a simulation of a network structure consisting of 250 nodes. Initially, each of these nodes could take on 1 of the 5 states. Threshold values were randomly assigned to individual nodes from a set of available values of 25%, 50%, 75% and 100%. The introduction of the threshold value (sensitivity parameter) therefore causes the mechanism to stabilise. Changes in the states of individual nodes are much slower. Both approaches are discussed in detail in [15]. An order parameter was also introduced, which clearly shows the process of phase transition:

(6) $$M=\sum_{i=1}^{k}\left|N_{s_{i}}-\frac{N}{k}\right|,$$

where *N* is the number of all nodes in the network structure, $N_{s_{i}}$ is the number of nodes being in the *s_i_* state, and *k* is the number of all states in the network. The order parameter was used to present the phase of the structure/system at a given moment. Its value depends on the number of individual states in which individual nodes are located in the network structure. The frequency of its value changes may indicate the instability of the entire structure related to frequent changes in the states of individual nodes. The further sections of the article present the tracking of changes in the value of the ordering parameter in order to detect various types of anomalies under consideration. However, example values of the order parameter for the standard (classic) approach and approach with a threshold value are presented at this point. Corresponding to Figure 1c,d, the value of the order parameter is shown increasing to the maximum value, and then the system goes into a stable state (ordered). Of course, in the case of a mechanism that takes into account the threshold values, the transformation time in the unstable phase is longer and depends both on the number of nodes, the number of initial states and the values of the threshold parameters. It should be noted that in the cases under consideration, the connection structure does not change—only the states of individual nodes change.

Based on the operation of the mechanism using the Potts model, it can be concluded that the phase transition maps the dynamics of changes taking place in the network structure. However, this course in relation to the presented examples is in fact dependent on the state of the initial network structure and the number of different states assigned to nodes. However, in order to broaden the analysis of the phase transition in network structures, one should take into account the impact of anomalies occurring in these infrastructures on the course of the phase transition process itself, and thus on the functioning of the network structure. Therefore, this issue was subjected to further research, and the results are presented in the further part of the paper.

## 3. The Concept of Anomaly

Before the analysis of anomalies or disorders in the assumed Potts model, the very concept of this phenomenon of anomalies should be defined. Numerous definitions relating to various fields and areas of science and technology can be found in the literature [33,34]. For this research, it was assumed that:

Anomalies or disorders in the network structure *G* = *{V,E}* are the set $A^{T}=\left\{a_{ij}^{\tau }:i=0,1,2,\ldots ;j=1,2,\ldots ,N;\tau \in T\right\},$ where $a_{ij}^{\tau }$ means a sudden change in state $s_{j}^{m}\to s_{j}^{n}$ of any node *v*_j_ in time *τ* going beyond the current trend determined by the value of the order parameter, whereby *T* denotes the analysed time interval, *i* denotes the identifier of the specific anomaly, *m* and *n* refer to state identifiers and N=|V|.

Taking into account the above definition, the classification of various types of anomalies that may appear in the network structure environment has been introduced.

- Homogeneous anomalies—these are disorders from the set *A*^T^, where $a_{ij}^{\tau }=a_{lk}^{\tau }$ for j≠k and ∀τ∈T. This disorder is of the same nature for the entire network structure and may occur at any time during the analysed time interval *T*.
- Heterogeneous anomalies—these are disorders from the set *A*^T^, where $a_{ij}^{\tau }\neq a_{lk}^{\tau }$ for j≠k as well as i≠l and ∀τ∈T. Thus, the disorder has a different character for different elements (in the case of the nodes in question) of the network structure. Typically, such disorders occur at different times of the analysed time interval *T*. However, it may be the case that heterogeneous anomalies occur at the same time for various elements of the network structure.
- Individual (single) anomalies—these are disorders occurring in the network structure from the set *A*^T^, where $a_{1j}^{\tau_{0}}$ for $\tau_{0}\in T$ and ∀j∈V, whereby $\sum_{j}=1$. It should be noted that this type of disorder usually has no major impact on the functioning of the network structure, and its cause may be a single error, failure, poor interpretation of results, etc.
- Group anomalies—these are disorders occurring in the network structure from the set *A*^T^, where $a_{ij}^{\tau_{}}$ for τ∈T and ∀j∈V, whereby $\sum_{j}>1$. A group disorder can occur both in one moment and also at a certain time interval.
- One-off anomalies—these are disorders that occur in the network structure from the set *A*^T^, where $a_{ij}^{\tau_{0}}$ for $\tau_{0}\in T$. They are disorders occurring once in the analysed time period, but they can cover more than one element of the network structure.
- Cyclic anomalies—these are disorders that occur in the network structure from the set *A*^T^, where $a_{ij}^{\tau_{c}}$ for c=1,2,…,C, and *C* means the number of cycles in a time interval *T*; therefore, C⊂T.

Considering the above definitions, it should be remembered that not every change in the state of a given element (node) of the network structure means an anomaly. If it results from the processes occurring in a given network structure, e.g., described by the Potts model, then such a change of state is not an anomaly. However, any unexpected changes that go beyond the current trend will be treated as a disorder.

## 4. Simulation Tests

A network structure consisting of 250 nodes was adopted to carry out simulation tests in the scope of the Potts model operation, taking into account anomalies. Each node could accept only one state from the set at a time $s=\left\{s_{i},i\in N\right\}$. During each iteration of the simulation, a node is randomly selected. The Hamiltonian for each of the available states in the vicinity of the node is then calculated. At the moment when the condition of changing the state of a given node in accordance with the adopted model is being fulfilled, such a change takes place. The condition for changing the current state of a given node is the predominance of another state in its environment. This activity is repeated for each node. In order to simulate anomalies, at the appropriate moment of the simulation, the corresponding function is activated, introducing a sudden change in the states of the nodes according to the rule corresponding to the specific type of anomaly. The forced change of individual nodes resulting from the anomaly is taken into account in subsequent simulation steps.

**Figure 2:**
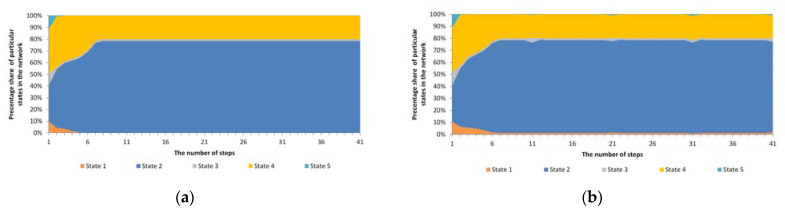

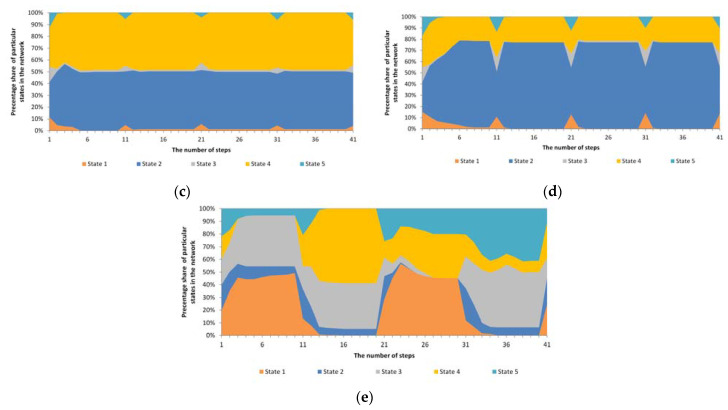
The course of operation of the standard Potts model along with cyclical anomalies for various numbers of nodes covered by anomalies (**a**) without anomalies; (**b**) 4% of nodes (**c**) 20% of nodes; (**d**) 50% of nodes; (**e**) 100% of nodes.

Figure 2 presents the simulation results, where a cyclical and heterogeneous anomaly with a different number of nodes covered by this anomaly was assumed, i.e., 4%, 20%, 50% and 100%. The mechanism was tested within 100 cycles, with anomalies being introduced 10 times in these 100 cycles. In order to verify the impact of the anomaly on the operation of the mechanism, the mechanism’s operation without anomalies was also taken into account. In all tests, the same initial distribution of individual states in the test network structure was adopted.

As shown in Figure 2, the operation of the Potts model without anomalies as well as with numerous cyclical anomalies proceeds in a similar direction. Once an anomaly has occurred, the system immediately stabilises in the distribution of individual states/opinions. As can be seen from the graphs presented, in this case, the unstable phase lasted for two simulation steps. As a result of the operation of this model, out of five initial states, three remained. However, the introduction of a cyclical anomaly forced the reappearance of these two vanished states during the simulation. Nevertheless, the trend of the model without anomalies and with anomalies has been the same. This is a result of the fact that even after an unforeseen change of state in a given node, the change in the next step resulted from the general impact of the surrounding environment. In order to be able to change such a trend, one should accept the principle that the changed state of a given node as a result of the anomaly will not be subject to further, normal effects of the Potts model.

**Figure 3:**
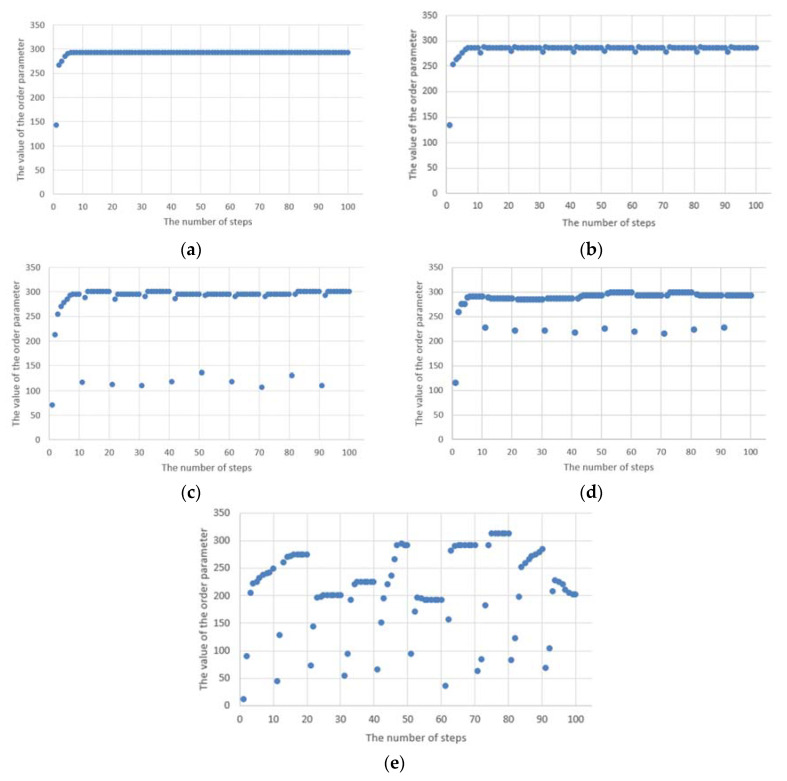
The values of the order parameter for the Potts model along with cyclical anomalies for different numbers of nodes covered by anomalies (**a**) without anomalies; (**b**) 4% of nodes (**c**) 20% of nodes; (**d**) 50% of nodes; (**e**) 100% of nodes.

However, taking into account phase transitions, it is necessary to analyse the order parameter for the simulation results presented above. Then, it can be observed that despite maintaining the proper trend of the Potts model (Figure 2), at the moment of the appearance of the anomaly, the network structure still goes into a disordered state, which is shown in Figure 3b–e. Of course, from the nature of the underlying Potts’s model, i.e., the influence of the surrounding environment on the state of the node, it follows that the structure in the case under consideration immediately returns to an orderly state. However, as long as there is an anomaly, the network structure cannot go to the stable (ordered) phase. Figure 3e shows that the time to reach a stable state is much longer. This is due to the fact that, in this case, all the nodes considered were covered by the anomaly. Moreover, this example shows clearly that the cyclic anomaly corresponds to the cyclic phase transitions.

Figure 3 shows that the value of the order parameter increases to the maximum value, and then, depending on whether there are anomalies or not, it enters the orderly state or remains in a state of disorder. In the latter case, as long as there are anomalies in the network structure, the phase transitions cannot take place. Moreover, the higher the anomaly ratio, the greater the discrepancy in the value of the order parameter. In the case of cyclical anomalies with an equal occurrence interval, one can notice point deviations from the ordering state and an immediate return to the orderly state. In the case when such an anomaly occurs for a short time, and then its appearance is distant in time, then it can be said that the time of convergence of the network structure (i.e., return to the stable work state) is short.

**Figure 4:**
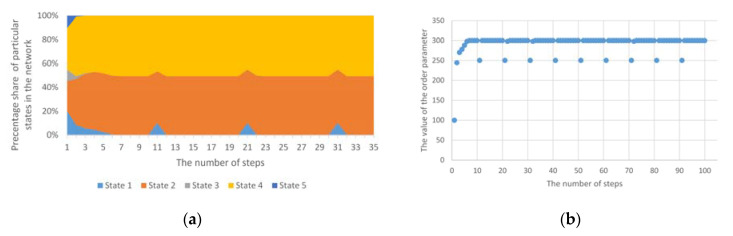
(**a**) The course of operation of the standard Potts model for cyclic homogeneous anomalies; (**b**) The values of the order parameter for the Potts model for a cyclic homogeneous anomaly.

The next considered case is the homogeneous cyclic anomaly. Changes in this anomaly refer to random *n* nodes, but change occurs to one specific state, regardless of the number of states available in the structure. The sample results are presented in Figure 4. In this case, a change was forced to state 1 in 10% of nodes. As can be seen, despite the temporary, sudden increase in the value of nodes in state 1, the system returns to its previously determined trend.

**Figure 5:**
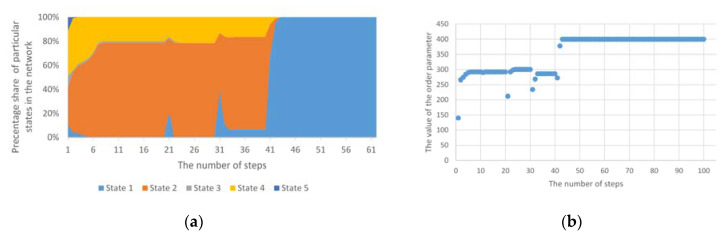
(**a**) The course of operation of the standard Potts model for cyclic homogeneous anomalies with the increasing number of nodes covered by the anomaly; (**b**) The values of the order parameter for the Potts model for a cyclic homogeneous anomaly with the increasing number of nodes covered by the anomaly.

Despite the maintained trend shown in Figure 4, it is possible to completely change the distribution of individual states as a result of a homogeneous anomaly. Such a change is possible after exceeding a certain limit of nodes, in which forced changes of, e.g., one state will appear. An example of this is shown in Figure 5. Then, the system is significantly affected by one of the states. Figure 5b also shows four phase transitions, and then full stability of the network structure appropriated by one state. Such anomalies may reflect real situations created in the structures of IT networks, e.g., as a result of malware or disinformation processes in social networks.

**Figure 6:**
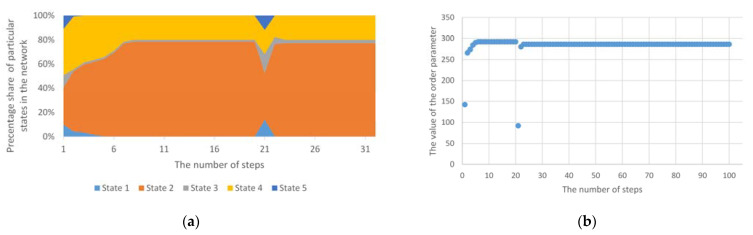
(**a**) The course of operation of the standard Potts model for a one-off, group anomaly; (**b**) The values of the order parameter for the Potts model for a one-off, group anomaly.

The phenomenon of an exemplary one-off group anomaly was presented, which covered 50% of nodes to give a broad perspective of various anomalies on the operation of the mechanism based on the Potts model in Figure 6. In this case, the changes included random nodes, and values of state changes were generated in a random manner. This anomaly did not cause long-term changes in the structure. Thus, the short-term external impact did not cause the nodes to remain in a modified state permanently. The environment had an effect on the anomalously affected nodes and thus they returned to a stable state consistent with the previous trend.

**Figure 7:**
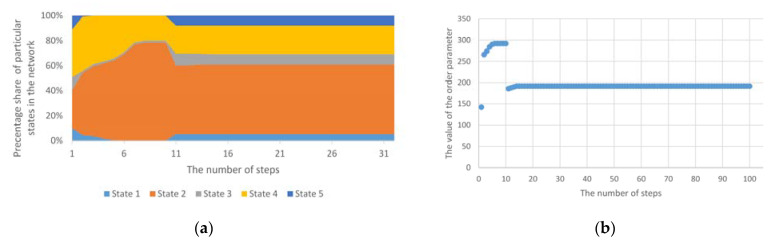
(**a**) The course of operation of the standard Potts model for homogeneous, group anomaly; (**b**) The values of the order parameter for the Potts model for homogeneous, group anomalies.

There may also be an anomaly which permanently forces a change in the state of given nodes. As a result of this disorder, the network structure from the stable state will go to the next stable state; then, we usually deal with a homogeneous multi-group anomaly. In this anomaly, there are several simultaneous groups of homogeneous anomalies. If, in the case of such an anomaly, it will not be accompanied by longer disturbances in the value of node states, then the convergence time may be omitted and then we do not talk about entering the unstable system phase again, but only a step change in the order parameter value. In this case, a completely new or previously eliminated state may appear (as in Figure 7a). One or more states that have stabilised earlier may also disappear. Such a situation will be visible in the achieved values of the order parameter (Figure 7b).

## 5. Examples of Applications

The introduction of anomalies or disorders to a mechanism based on the Potts model can be considered from two perspectives: firstly, taking into account unforeseen changes in the states of individual network nodes beyond the current trend; secondly, as a disruption in the phase change process. In the first case, we deal with a conscious or unconscious change in the value of these states. The reasons for these changes may vary—for example, a sudden change in opinion resulting from the emotions of a single person or a group of people in the social network structure (also conditioned by external factors), election spot, charity event, system error, appearance of malware in IT systems, congestion in computer network nodes caused by limited resources and damage to hardware or software, etc. The second perspective refers to the analysis of phase transition processes. As shown in Figure 1c,d, the order parameter clearly illustrates the phase change process. As a result of the simulations carried out on the basis of the Potts model, it can be stated that under normal conditions, i.e., without additional external disturbances, the network structure after the change (unstable) phase passes into the stabilised phase (ordered). This stabilised phase may last indefinitely. In contrast, the introduction of an anomaly to the mechanisms (either a group or a higher percentage factor) means that the system either cannot go to the ordered phase or returns to it after a certain time. By analysing this parameter, one can observe the dynamics of changes taking place in the structure of IT networks.

It seems that the analysis of phase transitions in the form of an analysis of changes in the value of the order parameter allows us to identify a specific type of anomaly. The exact estimation of the characteristics of the course of these changes for individual anomalies should be a separate study. Nevertheless, based on the results so far, it can be stated that each type of anomaly presented in this paper is characterised by a separate distribution of this parameter. The use of this property can be used in devices and systems, among others, to detect disturbances in computer networks, attacks on IT infrastructure and deliberate manipulation of behaviour (opinions) in social networks. For example, a homogeneous cyclic anomaly may correspond to interval tests of IT infrastructure before the actual DoS attack. However, in the case of this anomaly, with the increasing number of nodes covered by the anomaly, we can deal with the operation of malware or the operation of disinformation processes in social networks. Considering one-off anomalies, we can refer to individual disturbances resulting from transmission errors, errors in reading information from sensors or temporarily rejected packages as a result of congestion.

## 6. Conclusions

The aim of this paper was to present the possibilities of modelling various types of anomalies that may appear in the network structure, especially in IT, IoT and IoE networks. The causes of this type of phenomenon can be different. For example, in computer systems, this may be the result of malware, network interface failures, network bottlenecks or DoS attacks, and in social networks, the impact of external factors on the individual’s opinion or a conscious, independent decision that appears in spite of certain established trends as well as work disorders in the infrastructure of industry 4.0.

A mechanism based on the Potts model was used in the paper. Thanks to this, it was possible to simulate the impact of the surrounding environment on a given node. In addition, the introduction of the element of randomness into the mechanism allowed us to examine the behaviour and susceptibility of the entire structure to the occurrence of various types of anomalies. The classification of these anomalies was also presented, and then individual types of anomalies were simulated during the operation of the Potts model. The order parameter was also taken into account during the study. Thanks to this, it was possible to observe the dynamics of changes, as well as the processes related to the phase transitions accompanying the operation of the model itself, and the introduced anomalies. Due to the fact that the value of this parameter depends on the number of particular states in which particular nodes in the network structure are located, the frequency of its changes may indicate instability of the whole structure. Under normal conditions, the operation of the classical Potts model leads to the global ordering. On the other hand, the appearance of different types of anomalies affecting the change in state of individual nodes can significantly affect the change in the value of the order parameter. Therefore, by tracking the change in the value of this parameter, it is possible to observe and even diagnose a specific type of anomaly.

Summarising the results of tests and simulations, the impact of anomalies on phase transitions occurring in network structures has clearly been revealed. The obtained results can be used for the in-depth modelling of processes taking place in network structures, and, in particular, in IT, IoT and IoE networks of complex systems. Thanks to this, to increase the reliability and performance of the network’s industrial infrastructure, new network solutions may be developed in the future. Therefore, a target model of network disturbances implemented, e.g., in a simulation environment, can be built in the future.

## Data Availability

Data available on request due to restrictions.
